# Supplementary material for: Not every gene is special: Modelling scale controls the false discovery rate when analysing high-throughput sequencing data
Source: PLoS Comput Biol. 2026 Sep 8;22(9):e1014728. doi: 10.1371/journal.pcbi.1014728 (PMC13581218; doi:10.1371/journal.pcbi.1014728)
Supplement: Supplementary file 1 [file pcbi.1014728.s001.pdf]

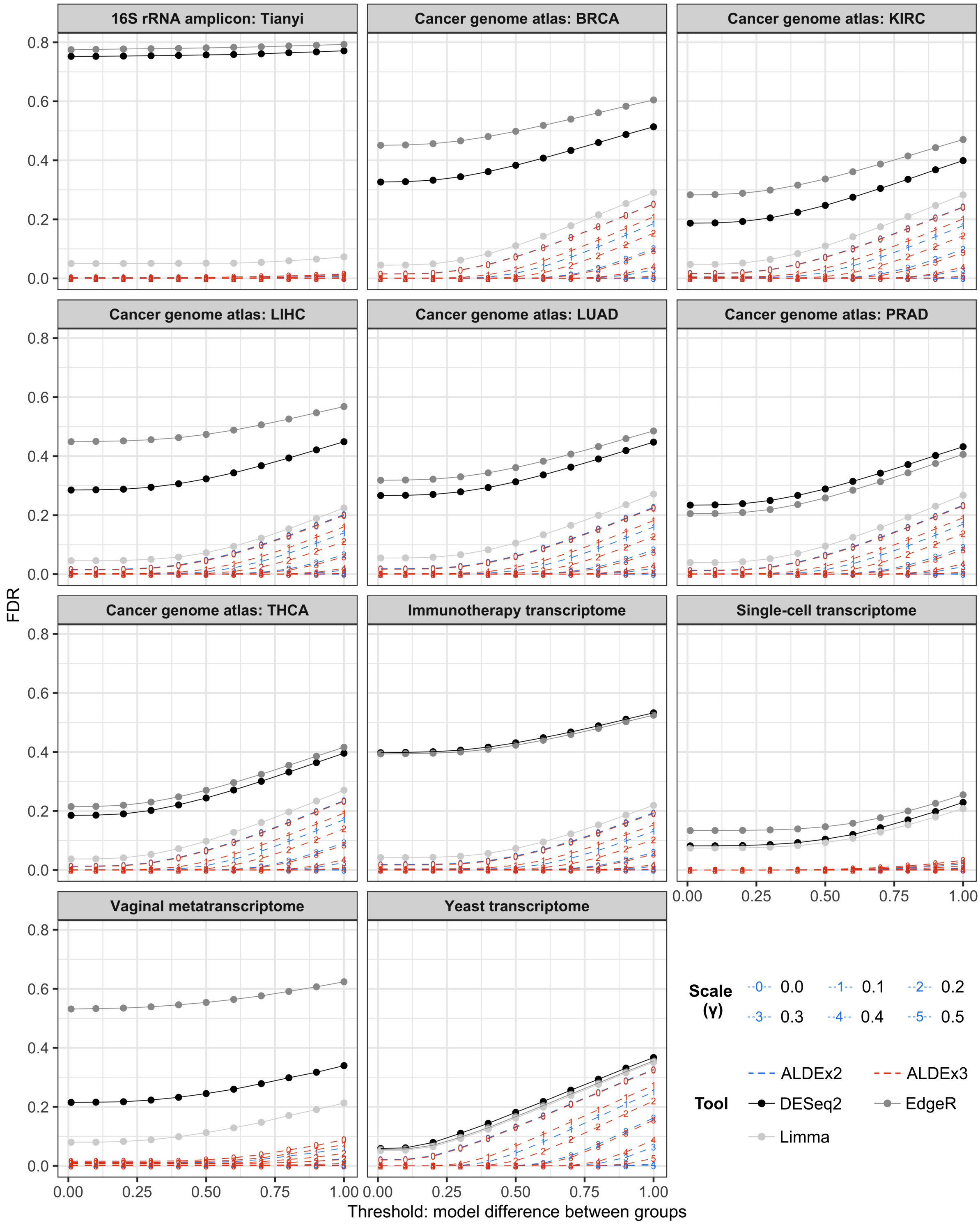

Supplementary Figure S1: **False-discovery rates for all datasets tested.** False-discovery rates (FDR) for all tool and dataset combinations were calculated separately for each 0.1 increment in the model difference between groups (i.e. the simulated or 'true' $\log_2$ fold-change between groups, as determined by binomial thinning). Each data point represents the FDR when considering dataset features with a simulated log-difference at or above the given x-axis threshold. Features were considered differentially abundant at a BH-adjusted P-value of <0.05.
