# Supplementary material for: Not every gene is special: Modelling scale controls the false discovery rate when analysing high-throughput sequencing data
Source: PLoS Comput Biol. 2026 Sep 8;22(9):e1014728. doi: 10.1371/journal.pcbi.1014728 (PMC13581218; doi:10.1371/journal.pcbi.1014728)
Supplement: Supplementary file 4 [file pcbi.1014728.s004.pdf]

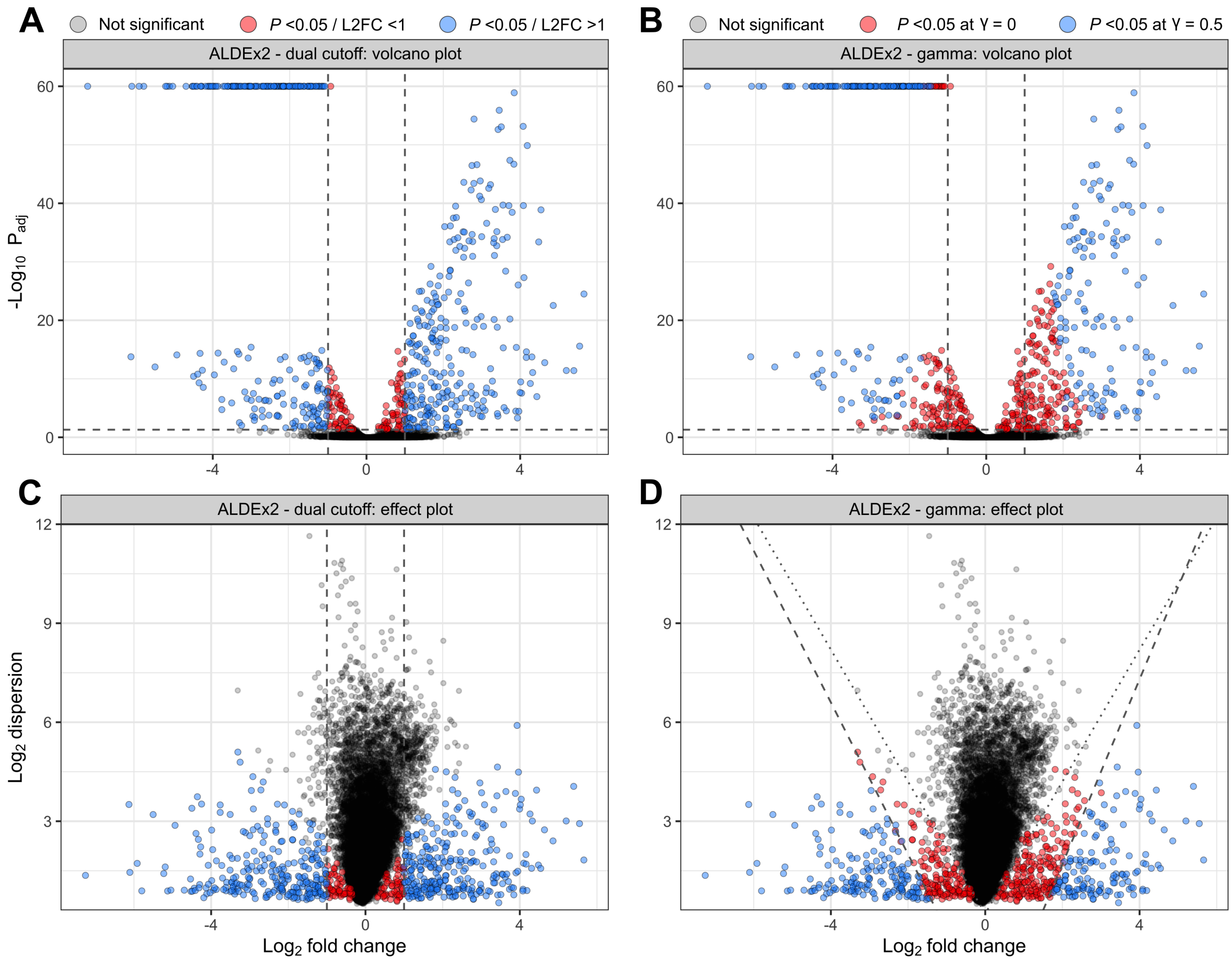

Supplementary Figure S4: **ALDEx2 scale models provide better FDR control than a dual *P*-value/fold-change cutoff.** A randomly selected analysis iteration of the PD1 immunotherapy dataset was used to create volcano plots (**A, B**) and effect plots (**C, D**) for ALDEx2 analyses. Each data point represents a feature from the scale-naïve analysis (γ = 0.001). Differentially expressed features are defined either by: a BH-adjusted *P*-value threshold of <0.05 *and* a fold-change cutoff of ≥1 (**A, B**); or a BH-adjusted *P*-value threshold of <0.05 when γ= 0.5 (**C, D**). Grey points indicate non-significant features. Coloured points indicate features which are significantly different between groups after applying different definitions of differential expression. Vertical dashed lines in **A-C** indicate a $\log_2$ fold-change of 1; horizontal dashed lines indicate BH-adjusted *P*-value <0.05. Grey dotted and dashed lines in **D** indicate approximate isarithms between non-significant and significant features when γ = 0.001 and 0.5, respectively.
