# Supplementary material for: Not every gene is special: Modelling scale controls the false discovery rate when analysing high-throughput sequencing data
Source: PLoS Comput Biol. 2026 Sep 8;22(9):e1014728. doi: 10.1371/journal.pcbi.1014728 (PMC13581218; doi:10.1371/journal.pcbi.1014728)
Supplement: Supplementary file 5 [file pcbi.1014728.s005.pdf]

Supplementary Table S1: **Average minimum log differences required for a significant result with ALDEx2 and ALDEx3 for all datasets and γ values.** For all datasets, the mean minimum log difference required for ALDEx2 and ALDEx3 to recognise a feature as differentially expressed (i.e. BH-adjusted P-value <0.05) was calculated for γ values ranging from 0 to 0.5. Data represent a mean of all 100 iterations of permuted/thinned HTS count data (TGCA, The Cancer Genome Atlas).

		Mean minimum difference (± s.d.)	
Dataset	Scale (γ)	ALDEx2	ALDEx3
Immunotherapy	0	**0.34** (0.045)	**0.36** (0.041)
	0.1	**0.54** (0.044)	**0.48** (0.040)
	0.2	**0.78** (0.059)	**0.64** (0.040)
	0.3	**1.02** (0.079)	**0.80** (0.051)
	0.4	**1.27** (0.108)	**0.98** (0.061)
	0.5	**1.52** (0.132)	**1.14** (0.070)
Metatranscriptome	0	**0.96** (0.261)	**0.96** (0.260)
	0.1	**1.10** (0.246)	**1.04** (0.257)
	0.2	**1.35** (0.280)	**1.21** (0.245)
	0.3	**1.59** (0.275)	**1.37** (0.271)
	0.4	**1.90** (0.327)	**1.57** (0.291)
	0.5	**2.16** (0.376)	**1.76** (0.291)
Single-cell	0	**0.85** (0.213)	**0.93** (0.250)
	0.1	**1.00** (0.202)	**1.01** (0.233)
	0.2	**1.21** (0.191)	**1.18** (0.230)
	0.3	**1.45** (0.199)	**1.34** (0.222)
	0.4	**1.73** (0.208)	**1.53** (0.225)
	0.5	**2.01** (0.253)	**1.72** (0.235)
TCGA	0	**0.24** (0.056)	**0.25** (0.055)
	0.1	**0.45** (0.051)	**0.39** (0.049)
	0.2	**0.69** (0.068)	**0.55** (0.053)
	0.3	**0.93** (0.089)	**0.72** (0.060)
	0.4	**1.17** (0.113)	**0.88** (0.068)
	0.5	**1.41** (0.140)	**1.05** (0.077)
Tianyi 16S rRNA	0	**1.81** (0.551)	**1.91** (0.552)
	0.1	**1.89** (0.523)	**1.96** (0.525)
	0.2	**2.07** (0.497)	**2.07** (0.549)
	0.3	**2.30** (0.518)	**2.19** (0.533)
	0.4	**2.50** (0.507)	**2.35** (0.519)
	0.5	**2.79** (0.684)	**2.61** (0.632)
Yeast	0	**0.18** (0.046)	**0.18** (0.046)
	0.1	**0.39** (0.045)	**0.32** (0.040)
	0.2	**0.63** (0.065)	**0.47** (0.044)
	0.3	**0.87** (0.087)	**0.65** (0.056)
	0.4	**1.11** (0.113)	**0.81** (0.062)
	0.5	**1.35** (0.147)	**0.98** (0.069)
