# Supplementary material for: Not every gene is special: Modelling scale controls the false discovery rate when analysing high-throughput sequencing data
Source: PLoS Comput Biol. 2026 Sep 8;22(9):e1014728. doi: 10.1371/journal.pcbi.1014728 (PMC13581218; doi:10.1371/journal.pcbi.1014728)
Supplement: Supplementary file 6 [file pcbi.1014728.s006.pdf]

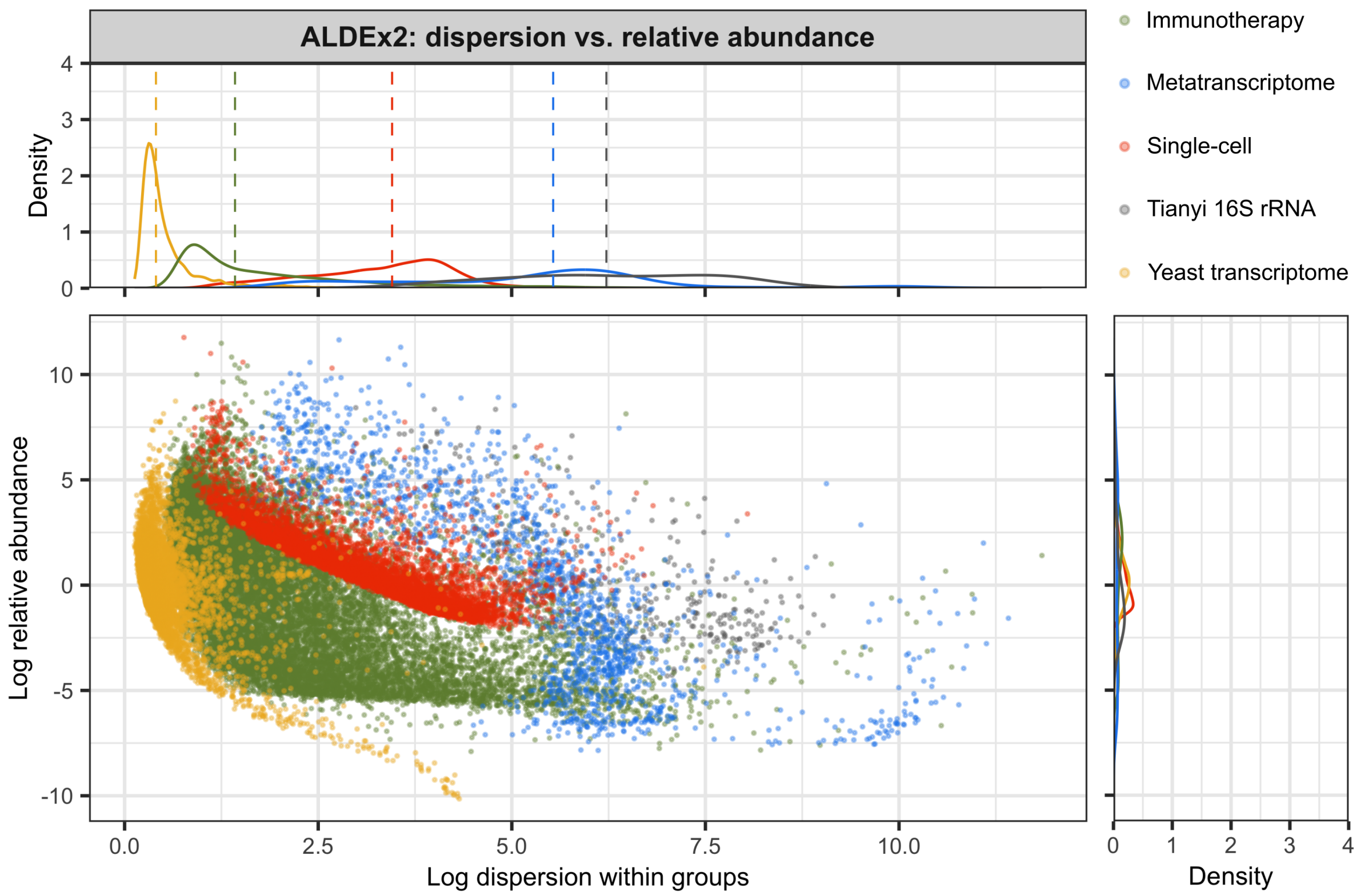

Supplementary Figure S5: **Features from 16S rRNA amplicons and metatranscriptome datasets have a larger dispersion compared to bulk RNA-seq data.** The within-group log dispersion of each feature was plotted against its log abundance. Data were sourced from a single, randomly selected iteration of the ALDEx2 permutation analyses from each dataset.
