# Supplementary material for: Not every gene is special: Modelling scale controls the false discovery rate when analysing high-throughput sequencing data
Source: PLoS Comput Biol. 2026 Sep 8;22(9):e1014728. doi: 10.1371/journal.pcbi.1014728 (PMC13581218; doi:10.1371/journal.pcbi.1014728)
Supplement: Supplementary file 7 [file pcbi.1014728.s007.pdf]

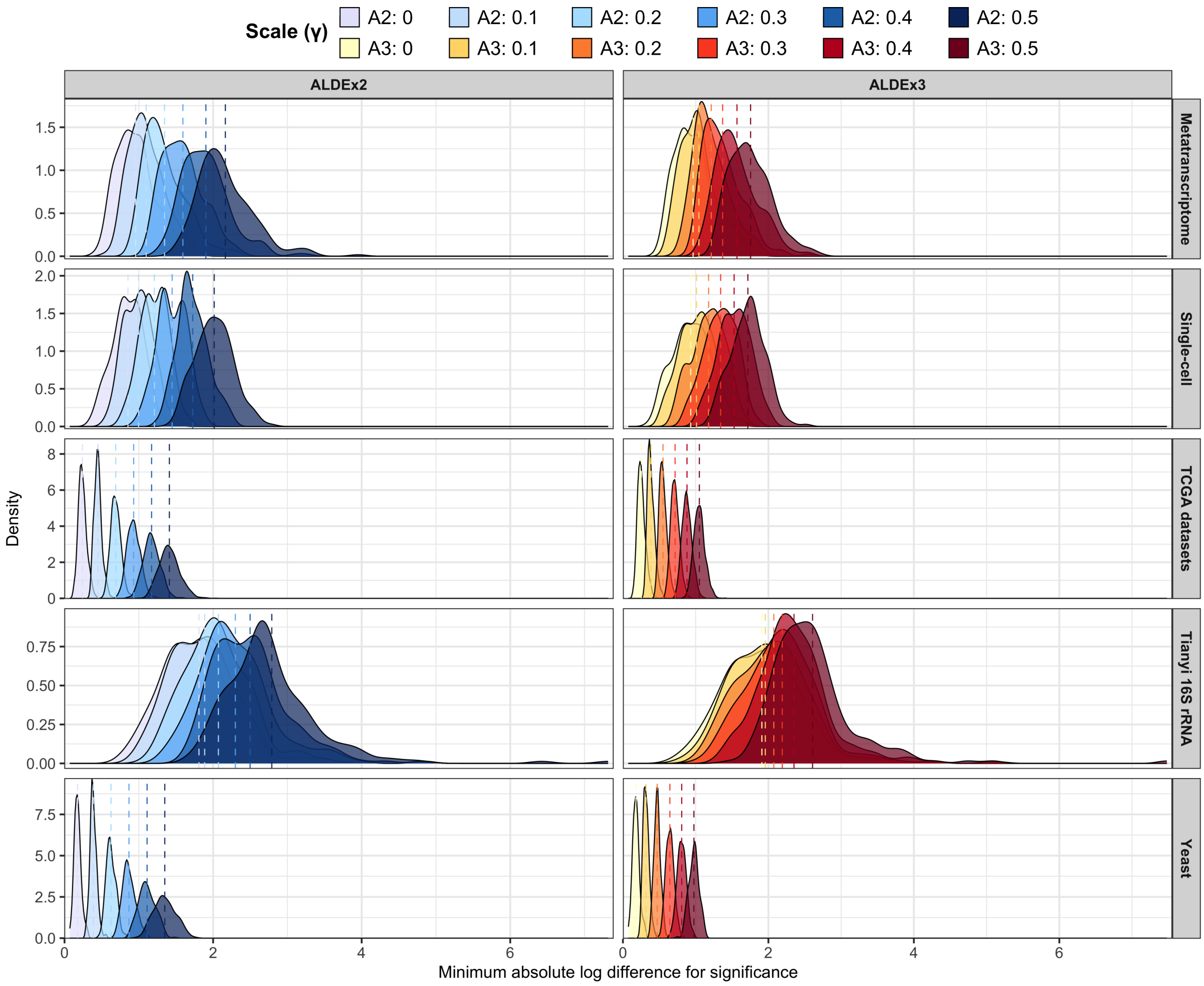

Supplementary Figure S6: **ALDEx3 returns a tighter distribution of minimum log-difference values required for significance across all datasets.** For each of the 100 iterations of the given dataset, the minimum absolute difference between groups needed for a gene to be reported as differentially expressed by ALDEx2 and ALDEx3 was recorded, based on a BH-adjusted *P*-value of <0.05, for γ values ranging from 0 to 0.5. Data for the Cancer Genome Atlas (TCGA), vaginal metatranscriptome, single-cell, 16S rRNA amplicon, and yeast transcriptome datasets are expressed as density plots and coloured by tool and γ value. Values for TCGA datasets represent an aggregate across the individual tumour transcriptome datasets. Dashed lines indicate the mean value.
