# Supplementary material for: Not every gene is special: Modelling scale controls the false discovery rate when analysing high-throughput sequencing data
Source: PLoS Comput Biol. 2026 Sep 8;22(9):e1014728. doi: 10.1371/journal.pcbi.1014728 (PMC13581218; doi:10.1371/journal.pcbi.1014728)
Supplement: Supplementary file 8 [file pcbi.1014728.s008.pdf]

Supplementary Table S2: **Differentially expressed genes reported by all tested tools for the original, non-permuted BRCA dataset:** The total number of genes identified as differentially expressed by ALDEx2 (A2), ALDEx3 (A3), DESeq2 (DS), edgeR (ER) and limma-voom (LM) are shown for increasing values of γ. At each γ value from 0.1 to 0.5, the number and proportion of genes belonging to the 'canonical drivers' and 'breast cancer' categories of the Network of Cancer Genes [1] that are no longer significantly different between groups (i.e. dropped genes) are also shown for ALDEx2 and ALDEx3. Data represent the results of a single analysis performed with the original, non-permuted count table and group membership vector. Dashes indicate non-applicable fields.

	No. differentially expressed genes					No. dropped NCG genes	
γ	A2	A3	DS	ER	LM	A2	A3
0	15,837	15,825	16,736	16,407	16,295	-	-
0.1	12,959	13,696	-	-	-	24 (0.83)	18 (0.85)
0.2	8,976	10,754	-	-	-	18 (0.45)	12 (0.41)
0.3	5,857	8,097	-	-	-	17 (0.55)	17 (0.64)
0.4	3,956	5,966	-	-	-	16 (0.84)	12 (0.56)
0.5	2,777	4,437	-	-	-	7 (0.59)	14 (0.92)

References

[1] Repana D, Nulsen J, Dressler L, Bortolomeazzi M, Venkata SK, Tourna A, et al. The Network of Cancer Genes (NCG): a comprehensive catalogue of known and candidate cancer genes from cancer sequencing screens. Genome Biology. 2019;20:1. doi:10.1186/s13059-018-1612-0.
