# Supplementary material for: Not every gene is special: Modelling scale controls the false discovery rate when analysing high-throughput sequencing data
Source: PLoS Comput Biol. 2026 Sep 8;22(9):e1014728. doi: 10.1371/journal.pcbi.1014728 (PMC13581218; doi:10.1371/journal.pcbi.1014728)
Supplement: Supplementary file 9 [file pcbi.1014728.s009.pdf]

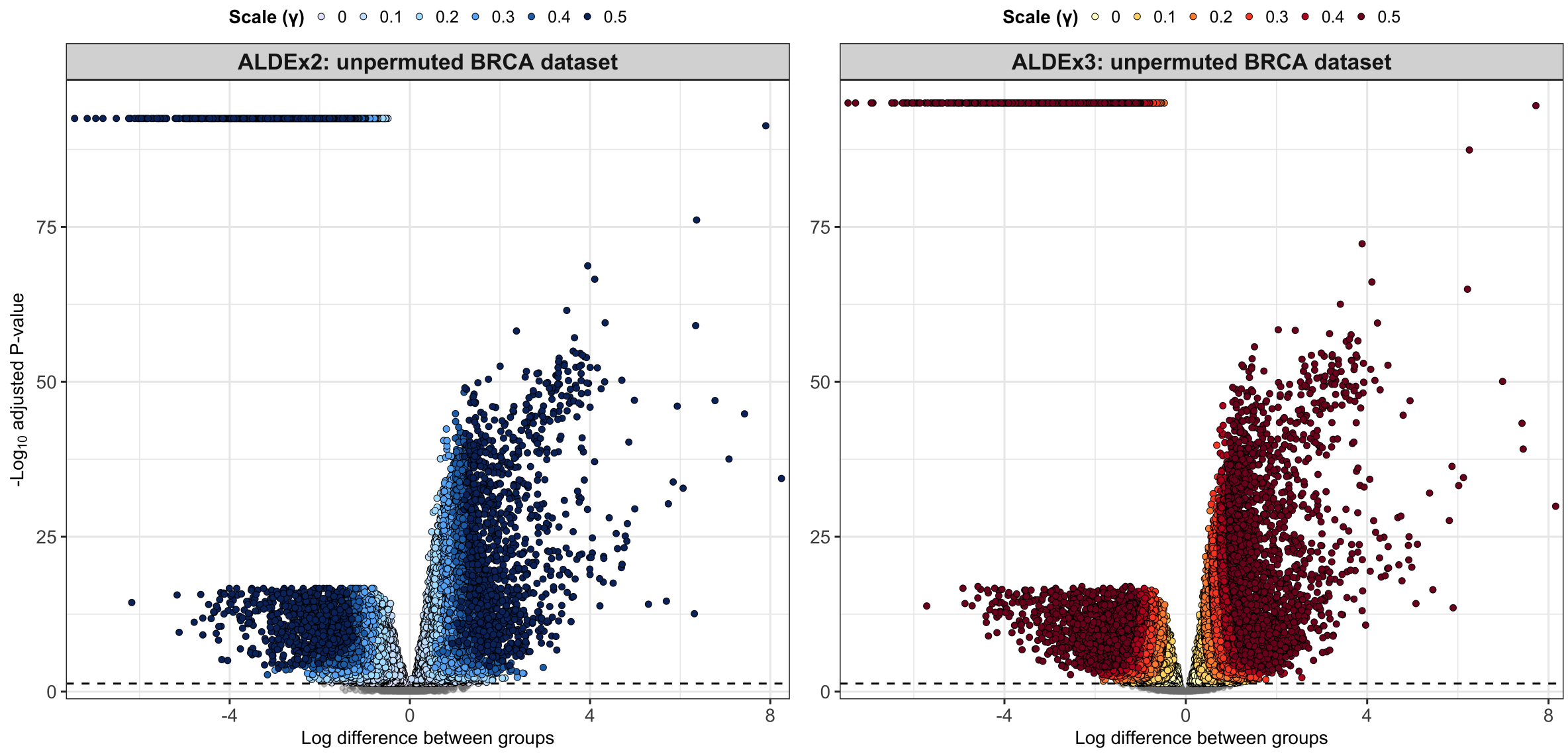

Supplementary Figure S7: **Boundaries between features reported as differentially expressed at different γ values are curved:** Volcano plots of log difference between groups vs. -$\log_{10}$ BH-adjusted P-values were generated for ALDEx2 (**left**) and ALDEx3 (**right**) analyses of the original, non-permuted BRCA dataset from the Cancer Genome Atlas. Curved boundaries between points identified as differentially expressed at different γ values can be clearly seen. Each data point represents a feature from the scale-naïve analysis ($\gamma = 0.001$). Grey points indicate non-significant features; coloured points indicate features which are significantly different after a certain amount of scale uncertainty has been added to the model. Grey dashed lines indicate BH-adjusted P-value <0.05.
