# Supplementary material for: Not every gene is special: Modelling scale controls the false discovery rate when analysing high-throughput sequencing data
Source: PLoS Comput Biol. 2026 Sep 8;22(9):e1014728. doi: 10.1371/journal.pcbi.1014728 (PMC13581218; doi:10.1371/journal.pcbi.1014728)
Supplement: Supplementary file 10 [file pcbi.1014728.s011.pdf]

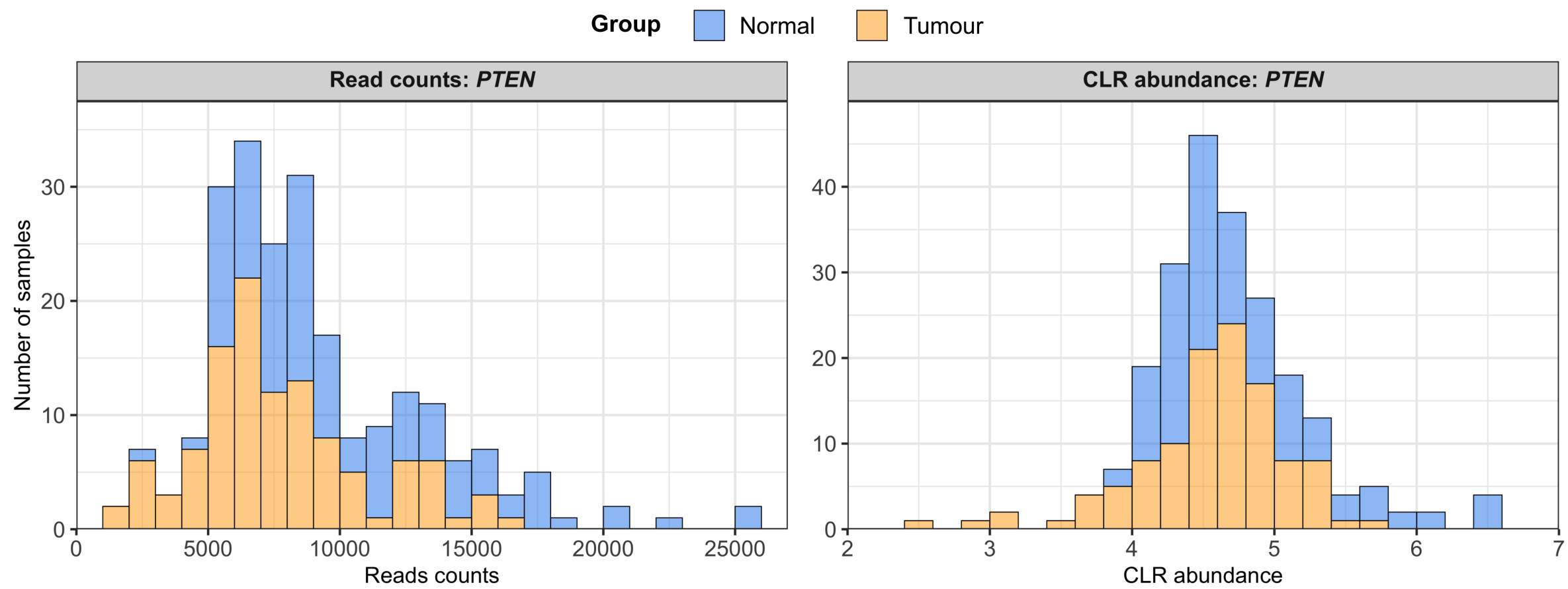

Supplementary Figure S8: **Significant differences in *PTEN* expression are driven by a few outlier samples:** Expression of the tumour suppressor gene, *PTEN*, in the Cancer Genome Atlas BRCA dataset is shown in histograms of read counts (**left**) and centred log-ratio (CLR) abundance values (**right**) calculated by ALDEx2. Bars are coloured by sample group. Bin width is 1,000 (reads) or 0.2 (CLR). The limited number of outlier samples in each group can clearly be seen at either end of the histograms, with most samples overlapping in the centre.
