# Supplementary material for: Not every gene is special: Modelling scale controls the false discovery rate when analysing high-throughput sequencing data
Source: PLoS Comput Biol. 2026 Sep 8;22(9):e1014728. doi: 10.1371/journal.pcbi.1014728 (PMC13581218; doi:10.1371/journal.pcbi.1014728)
Supplement: Supplementary file 11 [file pcbi.1014728.s012.pdf]

Review of "Not every gene is special: one simple rule to control the false discovery rate when analysing high-throughput sequencing data"

This paper presents a discussion of the strengths and limitations of current differential analysis tools, with a particular emphasis on the trade-off between sensitivity and power. The authors note that scale-based models, such as ALDEx2 and ALDEx3, are generally less sensitive than non-scaled approaches, but offer improved control of the false discovery rate (FDR). They further investigate how increasing the scale uncertainty parameter influences differential expression results within these frameworks. Additionally, through empirical experiments, the authors suggest that an appropriate choice of scale uncertainty can enable more reliable inference of log-fold changes for differential features. The reviewer appreciates the authors' effort to highlight key challenges in differential analysis; however, several aspects of the manuscript would benefit from further clarification and improvement.

1. The authors conclude, based on experimental results, that there exists an approximately linear relationship between L2FC and the scale uncertainty parameter (γ) in methods such as ALDEx2 and ALDEx3. They further suggest that setting ($\gamma = 0.2$) for ALDEx2 and ($\gamma = 0.3$) for ALDEx3 ensures that the false discovery rate (FDR) is controlled for inferred L2FC values greater than 0.5.

   However, this conclusion is derived from a limited set of 10 datasets and may not generalize broadly. It remains unclear whether the observed relationship holds across diverse real-world settings. As a result, the generalizability of this recommendation may require further justification. A more rigorous theoretical analysis would be highly valuable, rather than relying primarily on empirical observations from a small number of datasets.

2. In the experiments comparing the FDR control of different methods (Figure 1), the authors consider different thresholds for defining a feature as a true positive, which they refer to as the model difference. However, the conclusion that FDR control improves when the model difference is large may be questionable. When the model

difference threshold is set to a large value, some features with smaller effects may no longer be classified as positives simply because their differences do not exceed the chosen threshold, even though those differences may still be biologically meaningful. As a result, methods that detect such features may be penalized as having poor FDR control, even though they are identifying non-negligible differences that fall below the imposed cutoff. For this reason, it may not be fully appropriate to conclude that non-scale methods exhibit poor FDR control solely because they select features with effect sizes smaller than the specified threshold. This issue deserves further clarification.

3. In the abstract, the authors state that they performed "100 permutation analyses of 10 RNA-seq datasets." It would be helpful to clarify what is meant by "100 permutation analyses." Specifically, does this indicate that, for each dataset, the analysis was repeated 100 times using different permutations, and that performance metrics such as TPP and FDR were then averaged across these runs?

4. In lines 134-137, the authors state that "not all features with a modelled difference are actually detectable (i.e. false negatives, FNs)." However, according to lines 150-161, features whose magnitudes fall below the specified model difference threshold are classified as negatives. This appears to create a potential inconsistency in terminology.

5. In equation (3) and equation (5), the author uses $\mathbf{W}_{\cdot n}$, while in line 192, the author uses $\mathbf{W}_n$. The notation seems to be inconsistent and please revise.

6. In line 192, Y_n is not defined. Please revise.

7. In equation (5), since $Y_{\cdot,n}$ represents a vector, $\hat{\mathbf{W}}_{\cdot n}$ is also a vector based on the definition. But it seems that $\hat{\mathbf{W}}_{\cdot n}$ should be an estimator of total number of counts. Please revise or explain.

8. In line 196, the authors state that γ is drawn from a normal distribution with mean μ_n and variance Σ_n^2. However, in the implementation described in lines 199–207, μ_n and Σ_n^2 do not appear. It would be helpful to clarify how μ_n and Σ_n^2 are specified

in practice, and whether the parameter `gamma` controls the realized value of γ or the parameters of its distribution.

9. The authors include ten datasets in the analyses to provide more generalizable results. However, it is not clearly specified which datasets are used in each experiment. The authors are encouraged to provide these details to improve clarity and reproducibility.

10. The significance level used for FDR control in each experiment should be explicitly stated. Without this information, it is difficult to assess which methods achieve adequate FDR control.

11. The figures in the main text are not clearly presented, and it is difficult to distinguish between the lines corresponding to different γ values. The authors are encouraged to improve the resolution of the figures.

12. In Figure 2, the sensitivity of the scale-based models appears to fluctuate substantially across datasets. It would be helpful for the authors to explain the underlying reasons for this variability.

13. In the experiment that investigates the effect of increasing values of γ, it is unclear whether the features detected with smaller γ always fully include those detected with larger γ.

14. In Figure 3, it appears that when γ is large, only features with large differences between groups can be detected, and thus conclusion (ii) in line 285 is not immediately evident. Please provide further explanation.
