# Supplementary material for: Not every gene is special: Modelling scale controls the false discovery rate when analysing high-throughput sequencing data
Source: PLoS Comput Biol. 2026 Sep 8;22(9):e1014728. doi: 10.1371/journal.pcbi.1014728 (PMC13581218; doi:10.1371/journal.pcbi.1014728)
Supplement: Supplementary file 12 [file pcbi.1014728.s015.pdf]

Responses to reviewer comments

We thank both reviewers for reading our revised manuscript and for their most recent round of comments. We offer the following responses and/or justifications to the issues raised by reviewer #1 in blue text. We also wish to highlight a change to Figure 3, which has now been re-worked such that the same data is presented in a different way.

Reviewer 1

The revised manuscript has improved, but the major claims remain too broad and several methodological and clarity issues remain.

We thank reviewer #1 for their work on our manuscript which has definitely improved the overall quality. We address their comments below.

1. Regarding point 5 in the response letter, I do not find the authors' response satisfactory. The authors argue that the misapplication of tools to data types for which they were not originally designed is common. However, the fact that a practice is "common" does not justify its use in this manuscript. If the authors choose to apply DESeq2 or edgeR directly to cell-level single-cell RNA-seq data, they need to provide a clear methodological justification that the assumptions of these tools are appropriate in this setting, or provide additional sensitivity analyses, such as a pseudobulk-based comparison.

   We can appreciate why the reviewer did not find our previous response satisfactory and now offer a more robust explanation supported by new data. We agree that a pseudobulk approach would be helpful when discussing single-cell data and as such, we have now included one. The IFNB dataset was used by the authors of the popular Seurat package (>50,000 citations across all versions) in the publication accompanying v3 of their tool (https://doi.org/10.1016/j.cell.2019.05.031) and is used in the tutorial for their RNA-seq analysis pipeline (https://satijalab.org/seurat/archive/v3.2/immune_alignment.html). We have applied a pseudobulk approach using Seurat to analyse this dataset, covering 206 samples and ~14,000 genes.

   Once again, we observe the same phenomenon in Figures 1, 2, and 4B, showing that single-cell data analysed with a pseudobulk approach follow the same pattern as other datasets. Furthermore, this dataset shows a very similar relationship between the dispersion and abundance of its features compared to the previous single-cell dataset, albeit with somewhat more dispersion, as shown in Supplementary Figure 4) As with all other datasets, we provide all code and input files necessary to replicate this finding on GitHub.

2. I am also concerned by the authors' statement that "a quick search on Google" shows how often users apply these tools to such data. This is not an appropriate way to justify a methodological choice. Keyword co-occurrence, for example "single-cell RNA-seq" and "edgeR", does not necessarily mean that edgeR was used to analyse cell-level single-cell

data, nor does it demonstrate that such use is statistically appropriate. The authors should not rely on general keywords search results as evidence to support that their analysis strategy is valid.

We thank the reviewer for pushing us to be more precise in our claims- we are of course not trying to claim that the plural of ‘anecdote’ is ‘data’ by showing that one can find examples of the misapplication of tools to data on Google. Instead, we now include two additional key citations to support our assertion that bulk RNA-seq tools are commonly applied to single-cell data. We now cite "Confronting false discoveries in single-cell differential expression", by Squair et al. which shows that single-cell data suffers from similar false-discoveries in randomized data as does bulk-RNA seq (Li et al) and microbiome data (Skinnider). In particular, Figure 4D of this paper shows that many of these FD are small difference : low variance genes or high difference : high variance genes, precisely what is removed by adding a small amount of uncertainty to the normalisation. This paper also shows that the entire gamut of tools for differential expression analysis (including those used in our modelling and included in the seurat package) suffer from the high FDR problem.

A more recent benchmarking paper (Junttila et al: https://doi.org/10.1093/bib/bbac286) also shows that these tools are in common use in the single-cell field. Together, these two benchmarking papers indicate that bulk RNA-seq DA tools are common options. We believe that this shows that the approaches we are modelling in our submission are widely used in the scRNA sequencing field.

3. In addition, the authors acknowledge that the single-cell dataset has distributional characteristics that differ substantially from the other datasets, and they also state that they do not advocate the use of ALDEx2 or ALDEx3 for single-cell RNA-seq data analysis. However, they still use the single-cell dataset to support the claimed “universal” effect of scale uncertainty. I find this logic difficult to follow. If the authors consider this dataset atypical and do not recommend these tools for this setting, then the single-cell results should not be used to support broad general conclusions.

   We apologise for the confusion with this statement and agree it was contradictory and poorly explained: this statement was referring to applying the analysis tools to single-cell count data without pseudobulking, as was reflected in our previous single-cell dataset (now removed from this manuscript entirely). We have deleted this statement as our inclusion of a pseudobulk approach now also addresses the reviewer’s comment and aligns with the general consensus in the field that pseudobulk aggregation and analysis using canonical bulk RNA-seq analysis tools is the preferred way of analysing single-cell data.

4. The addition of the 16S rRNA amplicon sequencing dataset does not fully address the concern about generalisability of FDR control. This dataset has no defined ground truth for true and false positives. Therefore, it cannot directly evaluate FDR control in the same way as the RNA-seq simulation analyses. The authors state that the 16S dataset was

analysed through 21 pairwise comparisons among age groups. This analysis may show that the relationship between gamma and the minimum L2FC required for significance is observed in another data type. However, it does not demonstrate that the FDR recommendations generalise to 16S data, or to high-throughput sequencing data more broadly.

The reviewer is entirely correct in pointing this out. We have now applied our full thinning/randomisation approach to one of the pairwise comparisons (kindergarteners vs. middle-aged individuals). This subset of the Tianyi 16S rRNA amplicon dataset comprises 217 samples and ~1,100 taxa. With this new analysis, we were able to replicate our previous findings and have updated Figures 1, 2, and 4B accordingly, as well as all Supplementary Figures where appropriate.

5. I agree with Reviewer 2 that the terms “universal” and “any type of high-throughput sequencing data” are too broad, especially in the Abstract. The study includes a limited range of datasets, many of which are cancer-related bulk RNA-seq datasets. The additional data types are limited: the single-cell dataset remains methodologically problematic, and the 16S amplicon dataset does not provide ground truth for evaluating FDR control. This is not sufficient to support universal claims across all high-throughput sequencing data types. The authors should revise these statements to more cautious wording, such as “across the datasets tested here”.

   In accordance with our responses to comments 1 and 3, we now show that our findings do appear to be universal and irrespective of the type of high-throughput sequencing data. The addition of the pseudobulked single-cell RNAseq and 16S rRNA amplicon datasets as requested by the reviewer now provides a direct evaluation of FDR control. We have now removed both the methodologically problematic non-pseudobulked dataset and pairwise comparisons of the non-permuted/thinned 16S rRNA data.

6. There are still several textual, formatting, and citation issues that should be corrected before publication. For example, the manuscript still contains unresolved citations, including “ALDEx2 and ALDEx3 R packages [?, 19]” and “ALDEx3 is based on linear models and requires fewer computational resources while still implementing the Bayesian approach to modelling and analysis [?]”. There is also a spelling error in “Bayesian toolboxs”. In the Methods section, the authors state “A further seven bulk RNA-seq datasets originating from the Cancer Genome Atlas project…”, but only six TCGA tumour types are listed: BRCA, KIRC, LIHC, LUAD, PRAD, and THCA.

   We apologise for this oversight and thank the reviewer for catching this. We have now corrected the referencing issues, the typos, and the inconsistency in the number of datasets raised by the reviewer.

Reviewer 2

The clarification provided in the revised manuscript addresses my previous concerns. I don't have further comments.

We thank reviewer #2 for taking the time to read our manuscript again and for their comments on our initial draft. Together with the comments from reviewer 1, the manuscript is better having been through these revisions.
