# Supplementary material for: Not every gene is special: Modelling scale controls the false discovery rate when analysing high-throughput sequencing data
Source: PLoS Comput Biol. 2026 Sep 8;22(9):e1014728. doi: 10.1371/journal.pcbi.1014728 (PMC13581218; doi:10.1371/journal.pcbi.1014728)
Supplement: Supplementary file 13 [file pcbi.1014728.s016.pdf]

Responses to reviewer comments

The manuscript has improved substantially, and I appreciate the authors' additional analyses, especially the inclusion of a pseudobulk single-cell RNA-seq dataset and the application of the thinning/randomisation framework to the 16S rRNA amplicon dataset. However, I still think a few points need to be addressed before acceptance.

We thank reviewer #1 for reading our revised manuscript and have addressed their outstanding concerns as below:

1. I am still not fully convinced by the use of terms such as "universal" ("we identify universal guidance …") and "… for the analysis of any type of HTS data". The evidence still comes from a limited set of 11 datasets, including several related bulk RNA-seq cancer datasets, one pseudobulk (single-cell) dataset, one metatranscriptome dataset, one yeast dataset, and one 16S dataset. These additional analyses are useful and encouraging, but I do not think they are sufficient to claim that the findings are universal or apply to any type of HTS data. I suggest using more precise wording, such as "across the datasets tested here" or "across the evaluated HTS datasets in this study".

   We have removed all claims of universality from the results and discussion section and have replaced according to the reviewer's suggestion. The wording now makes explicit mention that all findings are applicable to the datasets tested in this study.

2. The manuscript still contains inconsistent descriptions of the 16S analysis. In the response letter, the authors state that they have now applied the full thinning/randomisation approach to the 16S dataset and removed the previous non-permuted/non-thinned pairwise analysis. However, the Figure 4 legend still refers to "all 21 pairwise comparisons" from the "non-permuted 16S rRNA amplicon sequencing dataset". Similar wording also appears in Table 2 and the Supporting Information, including Table S1, which still refers to "all 21 pairwise combinations of non-permuted, non-thinned 16S rRNA amplicon sequencing data". This is confusing because it is no longer clear whether the results shown in Figure 4, Table2, and the Supporting Information are based on the old non-permuted pairwise analysis or the new thinning/randomisation analysis. The authors should carefully check Figure 4, Table 2, and all supplementary figure/table legends so that they consistently describe the final analysis actually used in the manuscript.

   We apologise to the reviewer for missing these corrections that should have been included in the previous revision! We have corrected all main text and supplementary material figure legends and table captions. We have also removed a statement in the main text referring to non-permuted 16S rRNA amplicon data when discussing figure 4B, as this is no longer included in the article.
