# Supplementary material for: Not every gene is special: Modelling scale controls the false discovery rate when analysing high-throughput sequencing data
Source: PLoS Comput Biol. 2026 Sep 8;22(9):e1014728. doi: 10.1371/journal.pcbi.1014728 (PMC13581218; doi:10.1371/journal.pcbi.1014728)
Supplement: Supplementary file 14 [file pcbi.1014728.s014.docx]

We thank the reviewers for their in-depth comments on our manuscript. Accompanying our revised manuscript, we provide the following responses to the points raised in blue text. For transparency, we have also uploaded these responses to this study’s GitHub (<https://github.com/amurariu/usri/blob/main/paper/PLoS/responses_to_reviewers.pdf>).

**Reviewer #1**

1. I suggest the authors tone down the title. The manuscript does not establish a universal “rule” for FDR control across HTS analyses. Rather, it demonstrates improved FDR behaviour under a specific simulation framework involving scale uncertainty and binomial thinning. The claims appear broader than what is formally supported.

We have changed the title of the manuscript to be more in line with the reviewer’s recommendation.

2. The Introduction has substantial space to discuss ALDEx2 and ALDEx3 (which is currently available only as a preprint). In contrast, other approaches to FDR control are discussed only briefly or are not mentioned at all. This gives me an impression that the manuscript is structured primarily around promoting a specific methodological framework rather than providing a balanced evaluation of competing strategies. As the topic of this study is FDR control, why other approaches to FDR stabilisation are not mentioned at all (e.g., hierarchical modelling, spike-in calibration - comparison to ERCC spike-in normalisation would be highly relevant). A more balanced positioning of the work within the broader methodological landscape would strengthen the manuscript.

The reviewer is correct to point this out and we have expanded both the introduction and discussion accordingly, adding information on other approaches to FDR control as requested.

3. The evaluation relies on permuting labels and injecting controlled fold changes via binomial thinning. This is statistically clean but biologically stylised. Real datasets may contain 1) global shifts in RNA content; 2) batch effects interacting with scale; 3) heteroskedastic variance structures, etc. So the current permutation strategy may not reflect real biological complexities.

This is a fair point to raise, but it is worth noting that binomial thinning was developed as a way to include some of these complexities when benchmarking as opposed to fully synthetic data that never includes them. However, to address the reviewer’s concern and to show generality, we have now added a further dataset from Bian et al. (2017), a 16S rRNA amplicon sequencing dataset originating from stool samples taken from >1,000 healthy Chinese individuals of 7 different age categories. For this dataset, we did not apply the permutation/binomial thinning approach used for the RNA-seq datasets. Rather, we subset the data into all 21 pairwise combinations of the age categories and ran ‘real’ differential abundance analyses using ALDEx2 and ALDEx3 using the same gamma values as the RNA-seq datasets. Critically, in Figure 4 we observed a slope that runs parallel with that of the RNA-seq datasets when modelling the effect of gamma on the minimum log-difference required for a significant result in ALDEx2 and ALDEx3. This demonstrates that the effect of gamma is 1) not a result of the permutation/thinning approach; and 2) appears to be universal across disparate types of sequencing data. We have updated Figure 4, Table 2, Supplementary Table S1 and Supplementary Figure S4 accordingly, as well as the corresponding methods and results sections.

4. I also have some questions regarding the handling of the single-cell RNA-seq dataset. First, restricting the analysis to 1000 randomly selected cells within the upper quartile of read counts per group may alter the original distributional structure of the data (if read depth distributions differ between groups).

We understand the reviewer’s hesitation here: the single-cell data is included as an additional dataset with very different distributional characteristics than that for any of the other datasets. In our revised manuscript, we now explicitly state this, along with the fact that we do not necessarily advocate for the analysis of single-cell data using ALDEx2 and point the reader to literature regarding the ongoing debate in the single-cell field regarding bespoke single-cell analysis tools vs pseudobulking approaches. We further believe that the inclusion of the 16S rRNA sequence data, and the observation that it behaves the same as the other types of high-throughput sequencing data, should help allay some of the reviewer’s concern that the single cell data is atypical in its response to the addition of scale.

5. Second, edgeR, DESeq2, and limma were originally developed for bulk RNA-seq analysis. While they can be applied to single-cell data under certain aggregation strategies (e.g. pseudobulk), it is not clear whether their use at the cell level is appropriate here. Given that different methods are designed for different data regimes, it would be important to clarify the intended scope of comparison and avoid drawing general conclusions about method performance outside their primary domains of applicability.

We wholeheartedly agree with the reviewer that these tools were designed for RNA-seq data and that they are often misapplied. However, the misapplication of these tools to data for which they have not been designed is unfortunately common (<https://doi.org/10.1007/978-1-4939-9240-9_25> ). A quick search on Google for ‘16S microbiome data DESeq2’ or ‘single cell RNA-seq edgeR’ shows how often users apply these tools to their data without checking if it is suitable, despite the maintainers of the tools cautioning against it (e.g. <https://support.bioconductor.org/p/9154606/>). This was part of our motivation for applying these tools in this manner: showing others the consequences doing so. We also include a reference to benchmarking studies (Nearing et al. 2022) that show the consequences of misapplication of these tools to 16S data. We appreciate that this was not stated clearly in our manuscript at any point and have now included the point raised by the reviewer in the introduction and discussion.

6. Expand the discussion of when scale modelling is most appropriate (e.g. tumour data, metatranscriptomics, global RNA shifts). To recap, the authors used 10 datasets, but most of them are tumour datasets (the PD1 immunotherapy dataset and six bulk RNA-seq datasets from the Cancer Genome Atlas project), one single-cell RNA-seq dataset (with the concerns noted above), a yeast transcriptome dataset, and a metatranscriptome dataset. In my view, the range of datasets may not be sufficiently representative to support broad general conclusions.

In accordance with the reviewer’s comment, we have elaborated on this in the discussion but for clarity, we recommend employing scale models in ALDEx2 or ALDEx3 regardless of the type of sequencing data being analysed. Additionally, we describe how to further adapt scale models in scenarios where there is a known difference in biological system scale between groups as measured by spike-ins or other external measurements.

The inclusion of an additional dataset further replicates our findings and expands our observations to an additional type of sequencing data (amplicon sequencing). Moreover, this new analysis reflects a ‘real-world’ analysis and not just permutations of semi-synthetic datasets. We also note that the data from the cancer genome atlas project originate from multiple different studies with vastly different study populations and demographics. Given that we are able to replicate our findings across these very different types of data and two different approaches, we believe our findings are sufficiently representative to support the general conclusions stated in the manuscript.

**Reviewer #2**

1. The authors conclude, based on experimental results, that there exists an approximately linear relationship between L2FC and the scale uncertainty parameter (*_γ_*) in methods such as ALDEx2 and ALDEx3. They further suggest that setting (*_γ_* _=0_*_._*_2_) for ALDEx2 and (*_γ_* _=0_*_._*_3_) for ALDEx3 ensures that the false discovery rate (FDR) is controlled for inferred L2FC values greater than 0.5. However, this conclusion is derived from a limited set of 10 datasets and may not generalize broadly. It remains unclear whether the observed relationship holds across diverse real-world settings. As a result, the generalizability of this recommendation may require further justification. A more rigorous theoretical analysis would be highly valuable, rather than relying primarily on empirical observations from a small number of datasets.

We thank the reviewer for their comment and, in response to them and reviewer #1, we now include an 11^th^ dataset from Bian et al. (2017), a 16S rRNA amplicon sequencing dataset originating from stool samples taken from >1,000 healthy Chinese individuals of 7 different age categories. For this dataset, we did not apply the permutation/binomial thinning approach used for the RNA-seq datasets. Rather, we subset the data into all 21 pairwise combinations of the age categories and ran ‘real’ differential abundance analyses using ALDEx2 and ALDEx3 using the same gamma values as the RNA-seq datasets. Using this approach, we were able to replicate our findings: Figure 4 shows the exact same pattern in this new 16S rRNA dataset as the various RNA-seq datasets analysed with our permutation/thinning approach, demonstrating that the effect of gamma appears to be universal, regardless of the type of sequencing data, and is not restricted to theoretical analyses using our permutation/thinning approach.

2. In the experiments comparing the FDR control of different methods (Figure 1), the authors consider different thresholds for defining a feature as a true positive, which they refer to as the model difference. However, the conclusion that FDR control improves when the model difference is large may be questionable. When the model difference threshold is set to a large value, some features with smaller effects may no longer be classified as positives simply because their differences do not exceed the chosen threshold, even though those differences may still be biologically meaningful. As a result, methods that detect such features may be penalized as having poor FDR control, even though they are identifying non-negligible differences that fall below the imposed cutoff. For this reason, it may not be fully appropriate to conclude that nonscale methods exhibit poor FDR control solely because they select features with effect sizes smaller than the specified threshold. This issue deserves further clarification.

We thank the reviewer for their comment on this issue and agree that we could have clarified our intended meaning better, which we have now addressed in the methods section of the revised manuscript. The main point that we wished to make with Figure 1 is that all of the scale-naïve tools – particularly DESeq2 and edgeR- identify many features as being significant when there are large differences between groups. Features that are identified by double filtering with marginally significant *P*-values and large fold changes could be true positives, or could be positive because of error in the measurement of fold change and will often not replicate (Ebrahmipoor et al. 2021: DOI 10.1093/bib/bbab053). The way we controlled for this was to pre-specify what the minimum fold-change was prior to analysis, so only those features with a fold-change larger than this will be detected. Thus, the scale-aware and scale naïve tools are being tested on a level playing field. We also note in our revised manuscript that the poor FDR control of the non-scale aware tools has been repeatedly found in multiple independent studies using different data sets and experimental designs.

3. In the abstract, the authors state that they performed “100 permutation analyses of 10 RNA-seq datasets.” It would be helpful to clarify what is meant by “100 permutation analyses.” Specifically, does this indicate that, for each dataset, the analysis was repeated 100 times using different permutations, and that performance metrics such as TPP and FDR were then averaged across these runs?

We apologise for the lack of in this part of the abstract, which was partially due to space constraints. The reviewer’s interpretation of our meaning is correct, and we have clarified this in the revised abstract.

4. In lines 134-137, the authors state that “not all features with a modelled difference are actually detectable (i.e. false negatives, FNs).” However, according to lines 150-161, features whose magnitudes fall below the specified model difference threshold are classified as negatives. This appears to create a potential inconsistency in terminology.

This was a poor wording choice on our part, and we thank the reviewer for pointing this out. Lines 134-137 are describing the scenario where a feature may have had its counts altered by binomial thinning (i.e. adding signal to some degree), but the feature ends up not being detected by a given tool as differentially abundant between groups, potentially due to the amount of signal being very low. Therefore, such a scenario would be akin to a false-negative.

Lines 150-161 describe how we have calculated FDR/TPR at each model difference threshold. At each 0.1 increment from 0 to 1, we are considering only the true positives which have a modelled (i.e. injected) fold change greater than the given model difference threshold. If a feature *has* been altered by thinning but the amount of difference between groups is *below* the threshold, it is not considered a false-negative *per se*, it is just not counted towards the number of ‘true positives’ in the calculation of the FDR or TPR at that particular model difference threshold.

The statements on lines 134-137 and those on 150-161 are both true and not contradictory, but we do acknowledge that they were not well explained in the initial manuscript. We have clarified our intended meaning in the relevant sections of the revised manuscript to make this clear to readers.

5. In equation (3) and equation (5), the author uses **_W_**_·_*_n_*, while in line 192, the author uses **_W_***_n_*. The notation seems to be inconsistent and please revise.

Apologies for this inconsistency- thank you for catching this. We have corrected this notation to W.n so that consistency is maintained.

6. In line 192, *_Yn_* is not defined. Please revise.

Thank you for pointing this out- we now define Y.n as the observed counts of all features in a given sample, n. We have also made the notation consistent, as in comment #5.

7. In equation (5), since *_Y_*_·_*_,n_* represents a vector, **_W_**ˆ _·_*_n_* is also a vector based on the definition. But it seems that **_W_**ˆ _·_*_n_* should be an estimator of total number of counts. Please revise or explain.

Apologies for the confusion here. W^.n represents the best estimate of both the true composition and true biological scale of sample n. W^.n is therefore comprised of the normalised (log-ratio) Dirichlet instances calculated from the observed count data, and also the modelled system scale.

8. In line 196, the authors state that *_γ_* is drawn from a normal distribution with mean *µ_n_* and variance. However, in the implementation described in lines 199–207, *_µn_* and _Σ_^2^*_n_* do not appear. It would be helpful to clarify how *_µn_* and _Σ_^2^*_n_* are specified in practice, and whether the parameter gamma controls the realized value of *_γ_* or the parameters of its distribution.

We thank the reviewer for pointing this out and agree it was not particularly clear as it was initially written. We now include a proper description of how Σ (now changed to lowercase σ) and µ are specified and what they represent in the methods section. Briefly, the gamma parameter of aldex.clr() controls the standard deviation (and by definition, the variance, σ^2^) of the Gaussian distribution that system scale values are drawn from. The mean of this distribution, µ, is set to 0 by default, but can be specified by the user when making a custom matrix of scale values using the command, ‘aldex.makeScaleMatrix()’.

9. The authors include ten datasets in the analyses to provide more generalizable results. However, it is not clearly specified which datasets are used in each experiment. The authors are encouraged to provide these details to improve clarity and reproducibility.

We apologise for the confusion and have improved the clarity when describing all results and in the figure legends. For the revised manuscript, we confirm that figures 1 and 2 refer to the corresponding RNA-seq datasets as per the titles above the plots, figure 3 and 4A refers only to the PD1 immunotherapy dataset, figure 4B refers to **all** datasets, including the new 16S rRNA amplicon sequencing dataset.

10. The significance level used for FDR control in each experiment should be explicitly stated. Without this information, it is difficult to assess which methods achieve adequate FDR control.

We thank the reviewer for raising this issue. We did state in the methods that each tool “must report a corresponding Benjamini-Hochberg (BH) adjusted *P*-value ≤0.05” to be considered a positive finding (lines 159 – 160 of original manuscript), but we have now added this information to all main text *and* supplementary figure legends for clarity.

11. The figures in the main text are not clearly presented, and it is difficult to distinguish between the lines corresponding to different *_γ_* values. The authors are encouraged to improve the resolution of the figures.

We apologise that there were issues with the resolution of the figures; however, this is likely outside of our control and could be related to compression of the figures by the journal’s review software. We provided high resolution (300 dpi) figures to the journal as per their requirements. The GitHub repository has very high resolution (600 dpi) figures of all figures and, while inconvenient, we direct the reviewer to the ‘figures’ directory if the issue persists with this revised manuscript. We will endeavour to make sure that resolution issues are resolved prior to publication.

12. In Figure 2, the sensitivity of the scale-based models appears to fluctuate substantially across datasets. It would be helpful for the authors to explain the underlying reasons for this variability.

We thank the reviewer for the opportunity to further clarify this issue. It is true that the sensitivity is quite variable for the ALDEx2/3 tools, and that this is exacerbated somewhat by including scale uncertainty. However, this needs to be interpreted along with the false positive rate in Figure 1, where the other tools have very high FP rates. It is trivial to have high sensitivity if everything is detected as being significant simply because every feature is seen as ‘winning the race’. We agree with the reviewer that the primary driver of new tools has been the belief that sensitivity is king, but the argument we are trying to make is that this is a poor metric. Rather, we should be trying to generate tools that excel at reproducibility with as much sensitivity as possible.

13. In the experiment that investigates the effect of increasing values of *_γ_*, it is unclear whether the features detected with smaller *_γ_* always fully include those detected with larger *_γ_*.

We apologise to the reviewer for this omission. They are correct that features detected with smaller gamma values always fully include those detected with larger gamma values and we have now clarified this in the results section where Figure 3 is presented.

14. In Figure 3, it appears that when *_γ_* is large, only features with large differences between groups can be detected, and thus conclusion (ii) in line 285 is not immediately evident. Please provide further explanation.

Increasing gamma values *prevents* features with small between group differences but minute *P*-values (i.e. potential false-positives) from being called as differentially abundant/expressed. For example, features which are reported as significant only when gamma is virtually zero but not when gamma is large are those that fit conclusion ii) on line 285 of the original manuscript. This is also in line with Figure 4B, whereby the minimum difference between groups required for a feature to be reported as differentially expressed increases as the value of gamma also increases. Note that the cutoff for considering which features are significant as scale uncertainty increases is a dynamic one, as shown in Figures 3 and Supplementary Figure S5.
