# Supplementary material for: Not every gene is special: Modelling scale controls the false discovery rate when analysing high-throughput sequencing data
Source: PLoS Comput Biol. 2026 Sep 8;22(9):e1014728. doi: 10.1371/journal.pcbi.1014728 (PMC13581218; doi:10.1371/journal.pcbi.1014728)
Supplement: Peer review history

# Peer review: Decision Letter 0

**DOI:** 10.1371/journal.pcbi.1014728.r001

**Authors:** Can Yang, Mark Ziemann

1 Apr 2026

Not every gene is special: one simple rule to control the false discovery rate when analysing high-throughput sequencing data

PLOS Computational Biology

Dear Dr. Gloor,

Thank you for submitting your manuscript to PLOS Computational Biology. After careful consideration, we feel that it has merit but does not fully meet PLOS Computational Biology's publication criteria as it currently stands. Therefore, we invite you to submit a revised version of the manuscript that addresses the points raised during the review process.

Please submit your revised manuscript by Jun 01 2026 11:59PM. If you will need more time than this to complete your revisions, please reply to this message or contact the journal office at ploscompbiol@plos.org. When you're ready to submit your revision, log on to https://www.editorialmanager.com/pcompbiol/ and select the 'Submissions Needing Revision' folder to locate your manuscript file.

Please include the following items when submitting your revised manuscript:

* A letter that responds to each point raised by the editor and reviewer(s). You should upload this letter as a separate file labeled 'Response to Reviewers'. This file does not need to include responses to formatting updates and technical items listed in the 'Journal Requirements' section below.

* A marked-up copy of your manuscript that highlights changes made to the original version. You should upload this as a separate file labeled 'Revised Manuscript with Track Changes'.

* An unmarked version of your revised paper without tracked changes. You should upload this as a separate file labeled 'Manuscript'.

If you would like to make changes to your financial disclosure, competing interests statement, or data availability statement, please make these updates within the submission form at the time of resubmission. Guidelines for resubmitting your figure files are available below the reviewer comments at the end of this letter

We look forward to receiving your revised manuscript.

Kind regards,

Mark Ziemann

Academic Editor

PLOS Computational Biology

Can Yang

Section Editor

PLOS Computational Biology

**Journal Requirements:**

If the reviewer comments include a recommendation to cite specific previously published works, please review and evaluate these publications to determine whether they are relevant and should be cited. There is no requirement to cite these works unless the editor has indicated otherwise.

1) Please ensure that the CRediT author contributions listed for every co-author are completed accurately and in full.

At this stage, the following Authors/Authors require contributions: Scott J. Dos Santos, Andreea C. Murariu, Justin D. Silverman, and Gregory B. Gloor. Please ensure that the full contributions of each author are acknowledged in the "Add/Edit/Remove Authors" section of our submission form.

The list of CRediT author contributions may be found here: https://journals.plos.org/ploscompbiol/s/authorship#loc-author-contributions

2) We ask that a manuscript source file is provided at Revision. Please upload your manuscript file as a .doc, .docx, .rtf or .tex. If you are providing a .tex file, please upload it under the item type u2018LaTeX Source Fileu2019 and leave your .pdf version as the item type u2018Manuscriptu2019.

3) Please provide an Author Summary. This should appear in your manuscript between the Abstract (if applicable) and the Introduction, and should be 150-200 words long. The aim should be to make your findings accessible to a wide audience that includes both scientists and non-scientists. Sample summaries can be found on our website under Submission Guidelines:

https://journals.plos.org/ploscompbiol/s/submission-guidelines#loc-parts-of-a-submission

4) Please amend your detailed Financial Disclosure statement. This is published with the article. It must therefore be completed in full sentences and contain the exact wording you wish to be published.

1) State what role the funders took in the study. If the funders had no role in your study, please state: "The funders had no role in study design, data collection and analysis, decision to publish, or preparation of the manuscript."

2) If any authors received a salary from any of your funders, please state which authors and which funders..

If you did not receive any funding for this study, please simply state: u201cThe authors received no specific funding for this work.u201d

**Reviewers' comments:**

Reviewer's Responses to Questions

**Comments to the Authors:**

**Please note here if the review is uploaded as an attachment.**

Reviewer #1: This manuscript addresses a longstanding issue in HTS analysis, namely the control of FDR under potential scale misspecification. The authors present a permutation-based benchmarking framework and argue that incorporating scale uncertainty (via ALDEx2/3) improves FDR control relative to commonly used tools. The topic is relevant and the study is technically substantial. However, I have several significant concerns regarding framing, methodological scope, benchmarking fairness, and generalisability of the conclusions. These issues do not invalidate the work but require substantial clarification and revision before the manuscript can be considered for publication.

1. I suggest the authors tone down the title. The manuscript does not establish a universal “rule” for FDR control across HTS analyses. Rather, it demonstrates improved FDR behaviour under a specific simulation framework involving scale uncertainty and binomial thinning. The claims appear broader than what is formally supported.

2. The Introduction has substantial space to discuss ALDEx2 and ALDEx3 (which is currently available only as a preprint). In contrast, other approaches to FDR control are discussed only briefly or are not mentioned at all. This gives me an impression that the manuscript is structured primarily around promoting a specific methodological framework rather than providing a balanced evaluation of competing strategies. As the topic of this study is FDR control, why other approaches to FDR stabilisation are not mentioned at all (e.g., hierarchical modelling, spike-in calibration - comparison to ERCC spike-in normalisation would be highly relevant). A more balanced positioning of the work within the broader methodological landscape would strengthen the manuscript.

3. The evaluation relies on permuting labels and injecting controlled fold changes via binomial thinning. This is statistically clean but biologically stylised. Real datasets may contain 1) global shifts in RNA content; 2) batch effects interacting with scale; 3) heteroskedastic variance structures, etc. So the current permutation strategy may not reflect real biological complexities.

4. I also have some questions regarding the handling of the single-cell RNA-seq dataset.

First, restricting the analysis to 1000 randomly selected cells within the upper quartile of read counts per group may alter the original distributional structure of the data (if read depth distributions differ between groups).

Second, edgeR, DESeq2, and limma were originally developed for bulk RNA-seq analysis. While they can be applied to single-cell data under certain aggregation strategies (e.g. pseudobulk), it is not clear whether their use at the cell level is appropriate here. Given that different methods are designed for different data regimes, it would be important to clarify the intended scope of comparison and avoid drawing general conclusions about method performance outside their primary domains of applicability.

5. Expand the discussion of when scale modelling is most appropriate (e.g. tumour data, metatranscriptomics, global RNA shifts). To recap, the authors used 10 datasets, but most of them are tumour datasets (the PD1 immunotherapy dataset and six bulk RNA-seq datasets from the Cancer Genome Atlas project), one single-cell RNA-seq dataset (with the concerns noted above), a yeast transcriptome dataset, and a metatranscriptome dataset. In my view, the range of datasets may not be sufficiently representative to support broad general conclusions.

Reviewer #2: The review is uploaded as an attachment.

**********

**Have the authors made all data and (if applicable) computational code underlying the findings in their manuscript fully available?**

The PLOS Data policy requires authors to make all data and code underlying the findings described in their manuscript fully available without restriction, with rare exception (please refer to the Data Availability Statement in the manuscript PDF file). The data and code should be provided as part of the manuscript or its supporting information, or deposited to a public repository. For example, in addition to summary statistics, the data points behind means, medians and variance measures should be available. If there are restrictions on publicly sharing data or code —e.g. participant privacy or use of data from a third party—those must be specified.

Reviewer #1: Yes

Reviewer #2: Yes

**********

PLOS authors have the option to publish the peer review history of their article (what does this mean?). If published, this will include your full peer review and any attached files.

If you choose “no”, your identity will remain anonymous but your review may still be made public.

**Do you want your identity to be public for this peer review?**  For information about this choice, including consent withdrawal, please see our Privacy Policy.

Reviewer #1: No

Reviewer #2: No

[NOTE: If reviewer comments were submitted as an attachment file, they will be attached to this email and accessible via the submission site. Please log into your account, locate the manuscript record, and check for the action link "View Attachments". If this link does not appear, there are no attachment files.]

**Figure resubmission:**

After uploading your figures to PLOS’s NAAS tool - https://ngplosjournals.pagemajik.ai/artanalysis, NAAS will process the files provided and display the results in the "Uploaded Files" section of the page as the processing is complete. If the uploaded figures meet our requirements (or NAAS is able to fix the files to meet our requirements), the figure will be marked as "fixed" above. If NAAS is unable to fix the files, a red "failed" label will appear above. When NAAS has confirmed that the figure files meet our requirements, please download the file via the download option, and include these NAAS processed figure files when submitting your revised manuscript.

**Reproducibility:**

To enhance the reproducibility of your results, we recommend that authors of applicable studies deposit laboratory protocols in protocols.io, where a protocol can be assigned its own identifier (DOI) such that it can be cited independently in the future. Additionally, PLOS ONE offers an option to publish peer-reviewed clinical study protocols. Read more information on sharing protocols at https://plos.org/protocols?utm_medium=editorial-email&utm_source=authorletters&utm_campaign=protocols

Attachment Submitted filename: review.pdf

# Author comment: Author response to Decision Letter 1

**DOI:** 10.1371/journal.pcbi.1014728.r002

29 May 2026

Attachment Submitted filename: responses_to_reviewers.docx

# Peer review: Decision Letter 1

**DOI:** 10.1371/journal.pcbi.1014728.r003

**Authors:** Can Yang, Mark Ziemann

12 Jun 2026

Not every gene is special: modelling scale controls the false discovery rate when analysing high-throughput sequencing data

PLOS Computational Biology

Dear Dr. Gloor,

Thank you for submitting your manuscript to PLOS Computational Biology. After careful consideration, we feel that it has merit but does not fully meet PLOS Computational Biology's publication criteria as it currently stands. Therefore, we invite you to submit a revised version of the manuscript that addresses the points raised during the review process.

Please submit your revised manuscript by Aug 12 2026 11:59PM. If you will need more time than this to complete your revisions, please reply to this message or contact the journal office at ploscompbiol@plos.org. When you're ready to submit your revision, log on to https://www.editorialmanager.com/pcompbiol/ and select the 'Submissions Needing Revision' folder to locate your manuscript file.

Please include the following items when submitting your revised manuscript:

* A letter that responds to each point raised by the editor and reviewer(s). You should upload this letter as a separate file labeled 'Response to Reviewers'. This file does not need to include responses to formatting updates and technical items listed in the 'Journal Requirements' section below.

* A marked-up copy of your manuscript that highlights changes made to the original version. You should upload this as a separate file labeled 'Revised Manuscript with Track Changes'.

* An unmarked version of your revised paper without tracked changes. You should upload this as a separate file labeled 'Manuscript'.

We look forward to receiving your revised manuscript.

Kind regards,

Mark Ziemann

Academic Editor

PLOS Computational Biology

Can Yang

Section Editor

PLOS Computational Biology

**Additional Editor Comments (if provided):**

**Journal Requirements:**

If the reviewer comments include a recommendation to cite specific previously published works, please review and evaluate these publications to determine whether they are relevant and should be cited. There is no requirement to cite these works unless the editor has indicated otherwise.

1) Please ensure that the CRediT author contributions listed for every co-author are completed accurately and in full.

At this stage, the following Authors/Authors require contributions: Scott J. Dos Santos, Andreea C. Murariu, Justin D. Silverman, and Gregory B. Gloor. Please ensure that the full contributions of each author are acknowledged in the "Add/Edit/Remove Authors" section of our submission form.

The list of CRediT author contributions may be found here: https://journals.plos.org/ploscompbiol/s/authorship#loc-author-contributions

**Reviewers' comments:**

Reviewer's Responses to Questions

**Comments to the Authors:**

**Please note here if the review is uploaded as an attachment.**

Reviewer #1: The revised manuscript has improved, but the major claims remain too broad and several methodological and clarity issues remain.

1. Regarding point 5 in the response letter, I do not find the authors’ response satisfactory. The authors argue that the misapplication of tools to data types for which they were not originally designed is common. However, the fact that a practice is “common” does not justify its use in this manuscript. If the authors choose to apply DESeq2 or edgeR directly to cell-level single-cell RNA-seq data, they need to provide a clear methodological justification that the assumptions of these tools are appropriate in this setting, or provide additional sensitivity analyses, such as a pseudobulk-based comparison.

I am also concerned by the authors’ statement that “a quick search on Google” shows how often users apply these tools to such data. This is not an appropriate way to justify a methodological choice. Keyword co-occurrence, for example “single-cell RNA-seq” and “edgeR”, does not necessarily mean that edgeR was used to analyse cell-level single-cell data, nor does it demonstrate that such use is statistically appropriate. The authors should not rely on general keywords search results as evidence to support that their analysis strategy is valid.

In addition, the authors acknowledge that the single-cell dataset has distributional characteristics that differ substantially from the other datasets, and they also state that they do not advocate the use of ALDEx2 or ALDEx3 for single-cell RNA-seq data analysis. However, they still use the single-cell dataset to support the claimed “universal” effect of scale uncertainty. I find this logic difficult to follow. If the authors consider this dataset atypical and do not recommend these tools for this setting, then the single-cell results should not be used to support broad general conclusions.

2. The addition of the 16S rRNA amplicon sequencing dataset does not fully address the concern about generalisability of FDR control. This dataset has no defined ground truth for true and false positives. Therefore, it cannot directly evaluate FDR control in the same way as the RNA-seq simulation analyses. The authors state that the 16S dataset was analysed through 21 pairwise comparisons among age groups. This analysis may show that the relationship between gamma and the minimum L2FC required for significance is observed in another data type. However, it does not demonstrate that the FDR recommendations generalise to 16S data, or to high-throughput sequencing data more broadly.

3. I agree with Reviewer 2 that the terms “universal” and “any type of high-throughput sequencing data” are too broad, especially in the Abstract. The study includes a limited range of datasets, many of which are cancer-related bulk RNA-seq datasets. The additional data types are limited: the single-cell dataset remains methodologically problematic, and the 16S amplicon dataset does not provide ground truth for evaluating FDR control. This is not sufficient to support universal claims across all high-throughput sequencing data types. The authors should revise these statements to more cautious wording, such as “across the datasets tested here”.

4. There are still several textual, formatting, and citation issues that should be corrected before publication.

For example, the manuscript still contains unresolved citations, including “ALDEx2 and ALDEx3 R packages [?, 19]” and “ALDEx3 is based on linear models and requires fewer computational resources while still implementing the Bayesian approach to modelling and analysis [?]”.

There is also a spelling error in “Bayesian toolboxs”.

In the Methods section, the authors state “A further seven bulk RNA-seq datasets originating from the Cancer Genome Atlas project…”, but only six TCGA tumour types are listed: BRCA, KIRC, LIHC, LUAD, PRAD, and THCA.

Reviewer #2: The clarification provided in the revised manuscript addresses my previous concerns. I don't have further comments.

**********

**Have the authors made all data and (if applicable) computational code underlying the findings in their manuscript fully available?**

The PLOS Data policy requires authors to make all data and code underlying the findings described in their manuscript fully available without restriction, with rare exception (please refer to the Data Availability Statement in the manuscript PDF file). The data and code should be provided as part of the manuscript or its supporting information, or deposited to a public repository. For example, in addition to summary statistics, the data points behind means, medians and variance measures should be available. If there are restrictions on publicly sharing data or code —e.g. participant privacy or use of data from a third party—those must be specified.

Reviewer #1: Yes

Reviewer #2: None

**********

PLOS authors have the option to publish the peer review history of their article (what does this mean?). If published, this will include your full peer review and any attached files.

If you choose “no”, your identity will remain anonymous but your review may still be made public.

**Do you want your identity to be public for this peer review?**  For information about this choice, including consent withdrawal, please see our Privacy Policy.

Reviewer #1: No

Reviewer #2: No

[NOTE: If reviewer comments were submitted as an attachment file, they will be attached to this email and accessible via the submission site. Please log into your account, locate the manuscript record, and check for the action link "View Attachments". If this link does not appear, there are no attachment files.]

**Figure resubmission:**

**Reproducibility:**

?>

# Author comment: Author response to Decision Letter 2

**DOI:** 10.1371/journal.pcbi.1014728.r004

31 Jul 2026

Attachment Submitted filename: responses_to_reviewers_2ndRound.pdf

# Peer review: Decision Letter 2

**DOI:** 10.1371/journal.pcbi.1014728.r005

**Authors:** Can Yang, Mark Ziemann

10 Aug 2026

PCOMPBIOL-D-25-02566R2

Not every gene is special: modelling scale controls the false discovery rate when analysing high-throughput sequencing data

PLOS Computational Biology

Dear Dr. Gloor,

Thank you for submitting your manuscript to PLOS Computational Biology. After careful consideration, we feel that it has merit but does not fully meet PLOS Computational Biology's publication criteria as it currently stands. Therefore, we invite you to submit a revised version of the manuscript that addresses the points raised during the review process.

Please submit your revised manuscript by Oct 10 2026 11:59PM. If you will need more time than this to complete your revisions, please reply to this message or contact the journal office at ploscompbiol@plos.org. When you're ready to submit your revision, log on to https://www.editorialmanager.com/pcompbiol/ and select the 'Submissions Needing Revision' folder to locate your manuscript file.

Please include the following items when submitting your revised manuscript:

* A letter that responds to each point raised by the editor and reviewer(s). You should upload this letter as a separate file labeled 'Response to Reviewers'. This file does not need to include responses to formatting updates and technical items listed in the 'Journal Requirements' section below.

* A marked-up copy of your manuscript that highlights changes made to the original version. You should upload this as a separate file labeled 'Revised Manuscript with Track Changes'.

* An unmarked version of your revised paper without tracked changes. You should upload this as a separate file labeled 'Manuscript'.

If you would like to make changes to your financial disclosure, competing interests statement, or data availability statement, please make these updates within the submission form at the time of resubmission. Guidelines for resubmitting your figure files are available below the reviewer comments at the end of this letter.

As the corresponding author, your ORCID iD is verified in the submission system and will appear in the published article. PLOS supports the use of ORCID, and we encourage all coauthors to register for an ORCID iD and use it as well. Please encourage your coauthors to verify their ORCID iD within the submission system before final acceptance, as unverified ORCID iDs will not appear in the published article. Only the individual author can complete the verification step; PLOS staff cannot verify ORCID iDs on behalf of authors.

We look forward to receiving your revised manuscript.

Kind regards,

Mark Ziemann

Academic Editor

PLOS Computational Biology

Can Yang

Section Editor

PLOS Computational Biology

**Reviewers' comments:**

Reviewer's Responses to Questions

**Comments to the Authors:**

**Please note here if the review is uploaded as an attachment.**

Reviewer #1: The manuscript has improved substantially, and I appreciate the authors’ additional analyses, especially the inclusion of a pseudobulk single-cell RNA-seq dataset and the application of the thinning/randomisation framework to the 16S rRNA amplicon dataset. However, I still think a few points need to be addressed before acceptance.

1. I am still not fully convinced by the use of terms such as “universal” (“we identify universal guidance …”) and “… for the analysis of any type of HTS data”. The evidence still comes from a limited set of 11 datasets, including several related bulk RNA-seq cancer datasets, one pseudobulk (single-cell) dataset, one metatranscriptome dataset, one yeast dataset, and one 16S dataset. These additional analyses are useful and encouraging, but I do not think they are sufficient to claim that the findings are universal or apply to any type of HTS data. I suggest using more precise wording, such as “across the datasets tested here” or “across the evaluated HTS datasets in this study”.

2. The manuscript still contains inconsistent descriptions of the 16S analysis. In the response letter, the authors state that they have now applied the full thinning/randomisation approach to the 16S dataset and removed the previous non-permuted/non-thinned pairwise analysis. However, the Figure 4 legend still refers to “all 21 pairwise comparisons” from the “non-permuted 16S rRNA amplicon sequencing dataset”. Similar wording also appears in Table 2 and the Supporting Information, including Table S1, which still refers to “all 21 pairwise combinations of non-permuted, non-thinned 16S rRNA amplicon sequencing data”. This is confusing because it is no longer clear whether the results shown in Figure 4, Table2, and the Supporting Information are based on the old non-permuted pairwise analysis or the new thinning/randomisation analysis. The authors should carefully check Figure 4, Table 2, and all supplementary figure/table legends so that they consistently describe the final analysis actually used in the manuscript.

**********

**Have the authors made all data and (if applicable) computational code underlying the findings in their manuscript fully available?**

The PLOS Data policy requires authors to make all data and code underlying the findings described in their manuscript fully available without restriction, with rare exception (please refer to the Data Availability Statement in the manuscript PDF file). The data and code should be provided as part of the manuscript or its supporting information, or deposited to a public repository. For example, in addition to summary statistics, the data points behind means, medians and variance measures should be available. If there are restrictions on publicly sharing data or code —e.g. participant privacy or use of data from a third party—those must be specified.

Reviewer #1: Yes

**********

PLOS authors have the option to publish the peer review history of their article (what does this mean?). If published, this will include your full peer review and any attached files.

If you choose “no”, your identity will remain anonymous but your review may still be made public.

**Do you want your identity to be public for this peer review?**  For information about this choice, including consent withdrawal, please see our Privacy Policy.

Reviewer #1: No

[NOTE: If reviewer comments were submitted as an attachment file, they will be attached to this email and accessible via the submission site. Please log into your account, locate the manuscript record, and check for the action link "View Attachments". If this link does not appear, there are no attachment files.]

**Figure resubmission:**

**Reproducibility:**

To enhance the reproducibility of your results, we recommend that authors of applicable studies deposit laboratory protocols in protocols.io, where a protocol can be assigned its own identifier (DOI) such that it can be cited independently in the future. Additionally, PLOS ONE offers an option to publish peer-reviewed clinical study protocols. Read more information on sharing protocols at https://plos.org/protocols?utm_medium=editorial-email&utm_source=authorletters&utm_campaign=protocols

# Author comment: Author response to Decision Letter 3

**DOI:** 10.1371/journal.pcbi.1014728.r006

11 Aug 2026

Attachment Submitted filename: responses_to_reviewers_3rdRound.pdf

# Peer review: Decision Letter 3

**DOI:** 10.1371/journal.pcbi.1014728.r007

**Authors:** Can Yang, Mark Ziemann

17 Aug 2026

Dear Dr. Gloor,

We are pleased to inform you that your manuscript 'Not every gene is special: modelling scale controls the false discovery rate when analysing high-throughput sequencing data' has been provisionally accepted for publication in PLOS Computational Biology.

Before your manuscript can be formally accepted you will need to complete some formatting changes, which you will receive in a follow up email. A member of our team will be in touch with a set of requests.

Please note that your manuscript will not be scheduled for publication until you have made the required changes, so a swift response is appreciated.

IMPORTANT: The editorial review process is now complete. PLOS will only permit corrections to spelling, formatting or significant scientific errors from this point onwards. Requests for major changes, or any which affect the scientific understanding of your work, will cause delays to the publication date of your manuscript.

Should you, your institution's press office or the journal office choose to press release your paper, you will automatically be opted out of early publication. We ask that you notify us now if you or your institution is planning to press release the article. All press must be co-ordinated with PLOS.

Thank you again for supporting Open Access publishing; we are looking forward to publishing your work in PLOS Computational Biology.

Best regards,

Mark Ziemann

Academic Editor

PLOS Computational Biology

Can Yang

Section Editor

PLOS Computational Biology

***********************************************************

Reviewer's Responses to Questions

**Comments to the Authors:**

**Please note here if the review is uploaded as an attachment.**

Reviewer #1: The authors have satisfactorily addressed my main concerns. I only noticed a typographical issue: “TGCA” in Table S1 should be “TCGA”, which can be handled during editorial/copyediting. I am happy to support acceptance.

**********

**Have the authors made all data and (if applicable) computational code underlying the findings in their manuscript fully available?**

The PLOS Data policy requires authors to make all data and code underlying the findings described in their manuscript fully available without restriction, with rare exception (please refer to the Data Availability Statement in the manuscript PDF file). The data and code should be provided as part of the manuscript or its supporting information, or deposited to a public repository. For example, in addition to summary statistics, the data points behind means, medians and variance measures should be available. If there are restrictions on publicly sharing data or code —e.g. participant privacy or use of data from a third party—those must be specified.

Reviewer #1: Yes

**********

PLOS authors have the option to publish the peer review history of their article (what does this mean?). If published, this will include your full peer review and any attached files.

If you choose “no”, your identity will remain anonymous but your review may still be made public.

**Do you want your identity to be public for this peer review?**  For information about this choice, including consent withdrawal, please see our Privacy Policy.

Reviewer #1: No

# Editor report: Acceptance letter

**DOI:** 10.1371/journal.pcbi.1014728.r008

**Authors:** Can Yang, Mark Ziemann

PCOMPBIOL-D-25-02566R3

Not every gene is special: modelling scale controls the false discovery rate when analysing high-throughput sequencing data

Dear Dr Gloor,

I am pleased to inform you that your manuscript has been formally accepted for publication in PLOS Computational Biology. Your manuscript is now with our production department and you will be notified of the publication date in due course.

The corresponding author will soon be receiving a typeset proof for review, to ensure errors have not been introduced during production. Please review the PDF proof of your manuscript carefully, as this is the last chance to correct any errors. Please note that major changes, or those which affect the scientific understanding of the work, will likely cause delays to the publication date of your manuscript.

Soon after your final files are uploaded, unless you have opted out, the early version of your manuscript will be published online. The date of the early version will be your article's publication date. The final article will be published to the same URL, and all versions of the paper will be accessible to readers.

For Research, Software, and Methods articles, you will receive an invoice from PLOS for your publication fee after your manuscript has reached the completed accept phase. If you receive an email requesting payment before acceptance or for any other service, this may be a phishing scheme. Learn how to identify phishing emails and protect your accounts at https://explore.plos.org/phishing.

Thank you again for supporting PLOS Computational Biology and open-access publishing. We are looking forward to publishing your work!

With kind regards,

Sharmila Kamatchi

PLOS Computational Biology | Carlyle House, Carlyle Road, Cambridge CB4 3DN | United Kingdom ploscompbiol@plos.org | Phone +44 (0) 1223-442824 | ploscompbiol.org | @PLOSCompBiol
